# In Situ Monitoring of Pulsed Laser Annealing of Eu-Doped Oxide Thin Films

**DOI:** 10.3390/ma14247576

**Published:** 2021-12-09

**Authors:** Michal Novotný, Jan Remsa, Šárka Havlová, Joris More-Chevalier, Stefan Andrei Irimiciuc, Sergii Chertopalov, Petr Písařík, Lenka Volfová, Přemysl Fitl, Tomáš Kmječ, Martin Vrňata, Ján Lančok

**Affiliations:** 1 Institute of Physics of the Czech Academy of Sciences, 182 21 Prague, Czech Republic; remsa@fzu.cz (J.R.); havlova@fzu.cz (Š.H.); morechevalier@fzu.cz (J.M.-C.); stefan.irimiciuc@inflpr.ro (S.A.I.); chertopalov@fzu.cz (S.C.); pisarik@fzu.cz (P.P.); volfl@fzu.cz (L.V.); fitlp@vscht.cz (P.F.); kmjec@fzu.cz (T.K.); lancok@fzu.cz (J.L.); 2 Department of Physics and Measurements, University of Chemistry and Technology, 166 28 Prague, Czech Republic; vrnatam@vscht.cz; 3 National Institute for Laser, Plasma and Radiation Physics (INFLPR), 077125 Măgurele, Romania; 4 Faculty of Science, Charles University, 128 40 Prague, Czech Republic

**Keywords:** pulsed laser deposition, pulsed laser annealing, zinc oxide, lutetium oxide, titanium oxide, europium, in situ monitoring, photoluminescence

## Abstract

Eu^3+^-doped oxide thin films possess a great potential for several emerging applications in optics, optoelectronics, and sensors. The applications demand maximizing Eu^3+^ photoluminescence response. Eu-doped ZnO, TiO_2,_ and Lu_2_O_3_ thin films were deposited by Pulsed Laser Deposition (PLD). Pulsed UV Laser Annealing (PLA) was utilized to modify the properties of the films. In situ monitoring of the evolution of optical properties (photoluminescence and transmittance) at PLA was realized to optimize efficiently PLA conditions. The changes in optical properties were related to structural, microstructural, and surface properties characterized by X-ray diffraction (XRD) and atomic force microscopy (AFM). The substantial increase of Eu^3+^ emission was observed for all annealed materials. PLA induces crystallization of TiO_2_ and Lu_2_O_3_ amorphous matrix, while in the case of already nanocrystalline ZnO, rather surface smoothening0related grains’ coalescence was observed.

## 1. Introduction

Eu^3+^-doped oxide thin films possess great potential for several emerging applications in optics, optoelectronics, and sensors, i.e., waveguides, display luminophores, imaging detectors, solar cells, and scintillators [1,2,3,4,5,6,7,8,9,10,11]. Eu^3+^ was the dopant of choice in relation to the previously mentioned applications due to its strong emission in the visible part of spectra centered at around 612 nm [12]. As an example of well-known Eu^3^ dopant host matrices we can mention semiconducting ZnO, TiO_2,_ and dielectric Lu_2_O_3_ oxides. The Eu^3+^-doped thin films are fabricated by a variety of methods [1,2], e.g., ion implantation [13], plasma-enhanced chemical vapor deposition [3,4,14], electrochemical deposition [5], hydrothermal deposition [15,16], chemical bath deposition [6], spraying [17,18], sputtering [4,7,8,19,20,21], evaporation [22,23], pulsed laser deposition (PLD) [24,25,26,27], matrix-assisted pulsed laser evaporation technique (MAPLE) [28], and sol-gel [9,10,29,30,31,32,33].

Post-deposition thermal treatment is usually required to activate the Eu ions and optimize the photoluminescence (PL) response [4,8,9,13,14,16,17,19,20,21,26,28,29,30,32,33]. However, the methods used for conventional thermal annealing require heating the samples to high temperature (>700 °C) for sufficiently long times in order for thermal diffusion of the defects to occur. The high-temperature processing limits the flexibility and practical applicability of these methods toward development of various optical and electro-optical devices. To address just the main disadvantages, only heat-resistant substrates can be used, undesirable crystallization of amorphous host might also occur, and, finally, the treatment of the entire sample may be undesirable in the device production.

The utilization of Pulsed Laser Annealing (PLA) to obtain and tune optically active Eu^3+^-doped films may profit from numerous advantages [34,35], i.e., (if necessary) horizontally structured local-rapid laser rating, ability to achieve ultra-high temperature in local scale, better controlled vertical thermal profile, controlled thermal profile, rapid processing, possibility of remote sample processing, and easy process automation. PLA plays, therefore, an important and challenging role in the development of *high-tech* electronic, optoelectronic, photonics, and sensing devices.

PLA technique offers a great flexibility in the number of control parameters available, i.e., laser wavelength, fluence (*F*_L_), repetition rate (*f*_rep_), number of shots, and ambient atmospheres [36,37,38]. Finding the optimal PLA conditions strongly depends on sample properties, i.e., film thickness, optical properties, and microstructure. Therefore, in order to find the optimal PLA conditions for maximizing Eu^3+^ PL response, a number of testing samples will be necessary. In situ monitoring of PLA can be an effective way to reach the optimal conditions accurately and fast.

In this paper, we report on in situ monitoring optical properties of Eu-doped ZnO, TiO_2_, and Lu_2_O_3_ thin films prepared by PLD within their processing by PLA using ArF excimer laser. Photoluminescence and optical transmittance measurement techniques were implemented. The effect of PLA on morphology, microstructure, and composition was evaluated.

## 2. Materials and Methods

ZnO:Eu, Lu_2_O_3_:Eu, and TiO_2_:Eu thin films were fabricated by PLD using Nd:YAG laser (λ = 266 nm, τ = 6 ns) and KrF laser (λ = 248 nm, τ = 20 ns). The targets were Eu_2_O_3_:ZnO (1% at. Eu), Eu_2_O_3_:Lu_2_O_3_ (3% at. Eu), and Eu_2_O_3_:TiO_2_ (1% at. Eu). Depositions were performed in an oxygen ambient. The vacuum chamber was pumped down to an ultimate pressure of 5.10^−4^ Pa. The films were grown on fused silica substrates maintained at room temperature. The distance between substrate and target was 50 mm. The deposition conditions are listed in Table 1.

**Figure 1:**
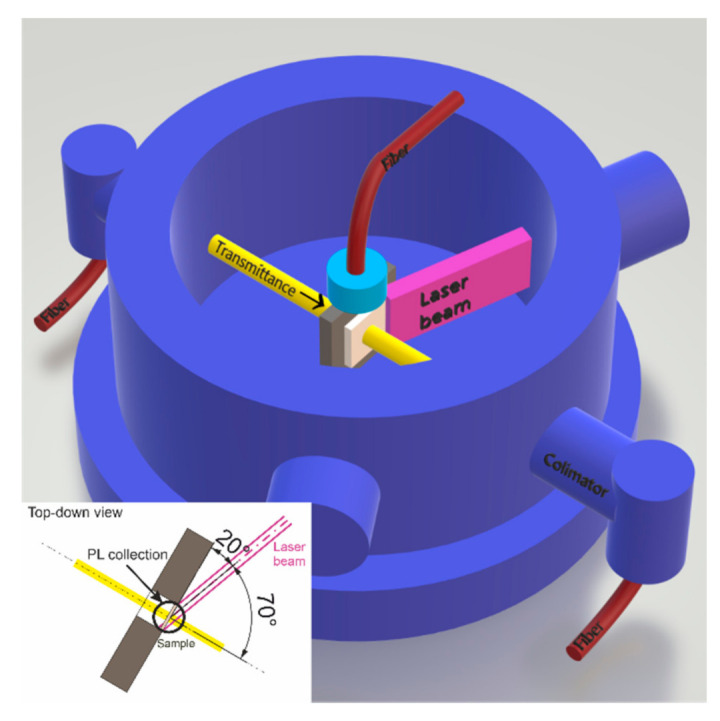
PLA and in situ optical properties’ measurement experimental setup.

PLA was performed using an ArF laser (λ = 193 nm, τ = 20 ns) Compex PRO 205 F (Coherent, Inc., Santa Clara, CA, USA). To perform PLA the laser beam was focused using converging lens on the sample at the angle of 70°. The optical properties were analyzed sequentially after each PLA pulse or burst of pulses that were fired at *f*_rep_ = 1 Hz. The signal for in situ PL was taken from the sample edge using an optical fiber connected to a spectrometer Horiba JY Triax iHR550 (Kyoto, Japan) equipped with LN cooled CCD Symphony. The same ArF laser was used for spectra excitation at lowered *F*_L_ = 10 mJ⋅cm^−2^ with *f*_rep_ = 5 Hz. Optical transmittance was in situ probed perpendicularly using light generated by a DH 2000 source (Ocean Insight, Orlando, FL, USA) and analyzed by a spectrometer. The PLA conditions are listed in Table 1 and the experimental setup is shown in Figure 1.

The structure of the films was characterized by X-ray diffraction (XRD) using an X-Ray diffractometer (EMPYREAN, Malvern Panalytical, Malvern, UK) with Cu anode (λ = 1.5406 Å) by grazing incidence (omega = 0.85°) and Bragg–Brentano measurements at room temperature. Morphology was examined by atomic force microscopy (AFM) (Dimension Icon, Bruker, Billerica, MA, USA). Chemical composition was determined by Scanning electron microscopy-Energy-dispersive X-ray spectroscopy (SEM-EDX) (Fera 3, TESCAN, Brno, Czech Republic) and the Orbis^®^ PC Micro-XRF spectrometer (EDAX, Ametek, Berwyn, PA, USA), Rh X-ray tube, 40 kV, polycapillary optics, a diameter of 30 µm of incident beam, and a silicon-drift detector with resolution FWHM~130 eV/Mn. The thickness measurement took place on an Alphastep IQ device (KLA-Tencor Corporation, Milpitas, CA, USA) with a scan length of 5 mm, stylus force of 5.5 mg, and scan speed of 50 µm·s^−1^. Each line was measured two times. The stylus diamond tip had a radius of 5 µm and an angle of 60°.

## 3. Results and Discussion

The deposited films of all investigated materials exhibited characteristic Eu^3+^ PL ^5^D_0_→^7^F_j_ (j = 0, 1, 2, 3, and 4) emission related to f-f transition. The observation of the PL emission confirmed that the energy transfer processes in Eu^3+^ excitation must take place considering excitation wavelength λ = 193 nm (6.42 eV). It can be mainly related to host absorption and marginally in case of Lu_2_O_3_:Eu also to the O^2−^–Eu^3+^ charge transfer band (CTB) [39,40]. Following hyper-sensitive electric dipole transition ^5^D_0_→^7^F_2_, its Stark splitting and ^5^D_0_→^7^F_2_/^5^D_0_→^7^F_1_ (magnetic dipole transition) emission ratio (asymmetry ratio), we could correlate the effect of PLA on the structural properties’ modification. It provided information related to the surrounding defects and disorder around the Eu^3+^ ions. The ^5^D_0_→^7^F_1_ transition was not considerably influenced by the surrounding of Eu^3+^ in the particular host. The transition ^5^D_0_→^7^F_2_ dominated all emission spectra from Eu^3+,^, suggesting the local symmetry around Eu^3+^ was low and deviated from an inversion center. The ^5^D_0_→^7^F_0_ transition was only allowed, taking into account the electric dipole selection rule, in the following 10 site symmetries: C_s_, C_1_, C_n_, and C_nv_ (n = 2, 3, 4, 6) [12].

The values of E_g_ listed in Table 2 were derived using Tauc plot—plotting (αhν)^1/m^ against photon energy (hν), where m is a parameter related to the type of transition, m = ½, and 2 corresponds to direct allowed transition and indirect allowed transition, respectively. TiO_2_ in anatase phase is considered as an indirect semiconductor, where m = 2 [41] while ZnO is a direct semiconductor (m = ½) [42] and Lu_2_O_3_ is considered as a direct ultrawide band gap semiconductor (m = ½) [43]. The E_g_ values were obtained with error of ±0.02 eV.

As for the chemical composition of the films, Eu dopant concentrations obtained by EDX and XRF analyses corresponded to those of the targets and any substantial variation after PLA was not detected.

In the following, let us discuss the properties’ modifications of each material, Eu-doped ZnO, TiO_2,_ and Lu_2_O_3_ by PLA, in more details.

### 3.1. ZnO:Eu

**Figure 2:**
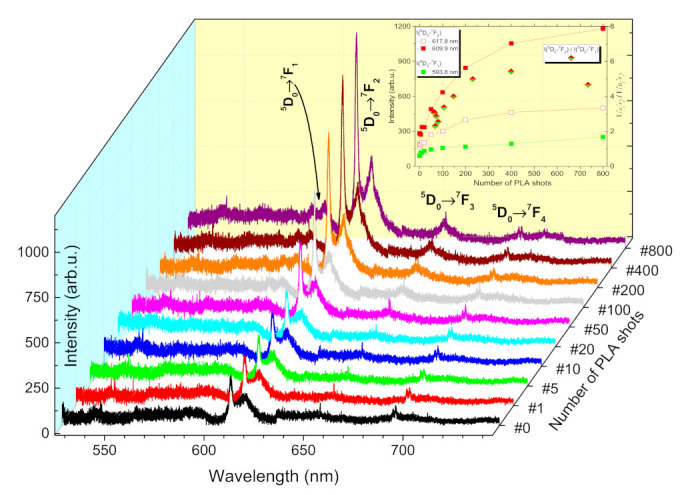
PL emission spectra of ZnO:Eu thin film, dependence on the number of PLA shots.

**Figure 3:**
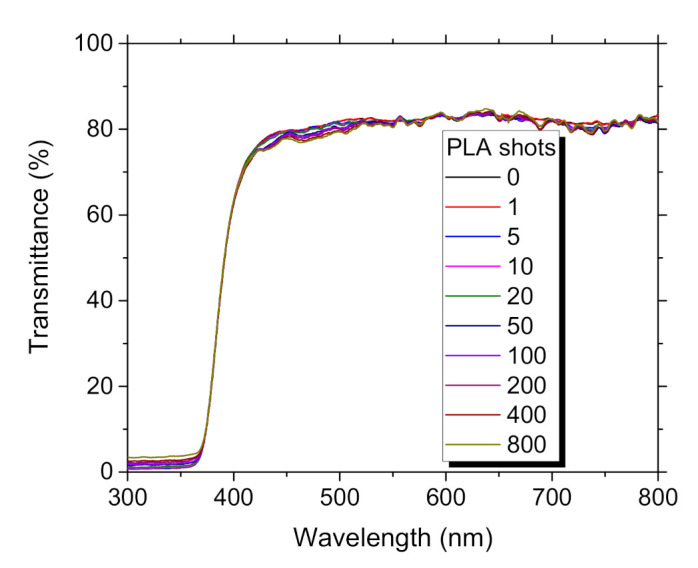
Transmittance spectra ZnO:Eu thin film, dependence on the number of PLA shots.

**Figure 4:**
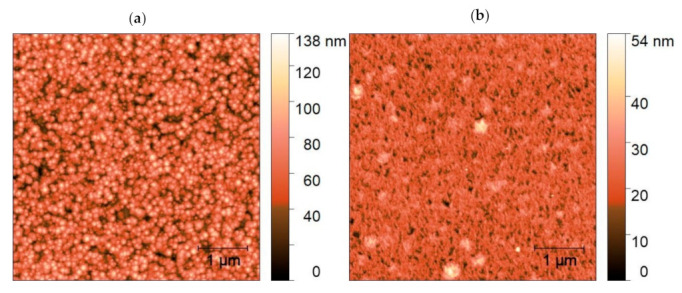
AFM surface images of ZnO:Eu thin film (**a**) as deposited and (**b**) after PLA processing, 800 shots.

The evolution of optical properties (PL emission and transmittance spectra) of ZnO:Eu films with increasing number of PLA shots is shown in Figure 2 and Figure 3, respectively. Characteristic Eu^3+^ red emission was detected at 587.3 nm and 593.8 nm (^5^D_0_→^7^F_1_), 609.9 nm and 618.8 nm (^5^D_0_→^7^F_2_), 654.8 nm (^5^D_0_→^7^F_3_), and 691.5 nm, 692.8 nm and 704.4 nm (^5^D_0_→^7^F_4_). The spectra in Figure 2 revealed that different emitting centers related to Eu may coexist in ZnO:Eu films, which is commonplace for wide band gap materials of wurtzite structure [44]. Eu^3+^ ions in ZnO:Eu films generally occupy the substitutional sites of Zn^2+^ ions. This fact turns ^5^D_0_→^7^F_1_ and ^5^D_0_→^7^F_2_ transitions to be allowed. The observation of ^5^D_0_→^7^F_2_ transition splitting suggests that Eu^3+^ can be at a substitutional Zn site (C_3v_ symmetry). We could not observe clear evidence of ^5^D_0_→^7^F_0_ transition whose appearance in PL spectra of ZnO:Eu was related with Eu^3+^ presence in other sites (e.g., the interstitial sites or surface/grain boundary of ZnO nanocrystals) [33,45,46]. A potential drawback of our interpretation can be the relatively low intensity of the PL signal. However, if we expect only a small fraction of Eu^3+^ in the sample located at ZnO nanocrystals’ surface, Eu^3+^ emission lines’ intensities and asymmetric ratio would increase with the increasing number of PLA shots, while the asymmetric ratio saturated at N_#_ = 400, as it is depicted in Figure 2. The enhancement of Eu^3+^ PL intensity at 609.9 nm by a factor of 4.1× was obtained. The PL signal intensity and asymmetric ratio variations might be related to microstructure modification, as displayed in Figure 4, where grains coalescing took place. It was reported that the energy relaxation process of excited Eu^3+^ ions should play a more important role toward PL efficiency enhancement than the energy transfer process from ZnO matrix to Eu^3+^ ions [47].

The transmittance spectra shown in Figure 3 confirm good quality of the film because transmittance of ~80% was reached in the visible light spectrum (VIS) band. There was not noticeable any variation in transmittance spectra, which confirmed that defects like oxygen vacancies were not introduced during the PLA processing. Band gap value decreased after PLA from E_g_~3.28 eV to 3.25 eV. Recently, we observed similar E_g_ values and trends at ZnO:Eu film PLD deposited at a substrate temperature of 300 °C [24].

AFM images of the film surface presented in Figure 4 revealed that PLA caused interconnecting of the grains accompanied by substantial lowering of surface roughness, as depicted in Table 2. We could attribute this effect to reaching the melting threshold of ZnO:Eu thin film. Similar results of UV PLA on the surface morphology of ZnO thin film of comparable thickness deposited by PLD on quartz substrate were reported at higher F_L_ ≥ 140 mJ/cm^2,^ where coalesced nanoclusters kept the wurtzite crystalline structure [36]. The PLA-induced coalescence of nanoparticles was also reported at ZnO [48] and ZnO:Eu [37] films of lower thickness < 60 nm on metal and Si substrates, respectively.

**Figure 5:**
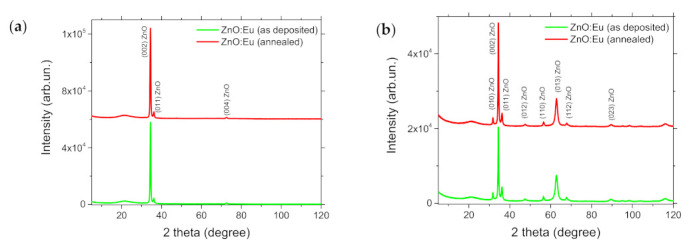
XRD patterns of ZnO:Eu thin film (as deposited and after PLA processing, 800 shots), (**a**) 2theta-omega and (**b**) grazing incidence scans.

XRD patterns of as-deposited and PLA-treated ZnO:Eu film, shown in Figure 5, did not reveal any substantial changes in structural properties. This fact is in good correlation with our previous results dedicated to PLA of ZnO:Eu film by KrF laser [37]. The film exhibited a hexagonal wurtzite structure of fibrous texture along the c-axes with random lateral orientations of crystallites. The FWHM of the strongest (002) peak was used to estimate the average crystallite size by using the Scherrer’s formula and were ≈19 nm. This correlated with the AFM measurement data. The presence of additional phases, such as Eu_2_O_3,_ was not detected. Considering the larger ionic radius of Eu^2+^ (1.13 Å) than Eu^3+^ (0.95 Å) in comparison with Zn^2+^ (0.74 Å), substitution of Eu^3+^ ions at Zn^2+^ sites seemed to be more favorable than that of Eu^2+^ ions but charge compensation must take place [24].

### 3.2. TiO_2_:Eu

**Figure 6:**
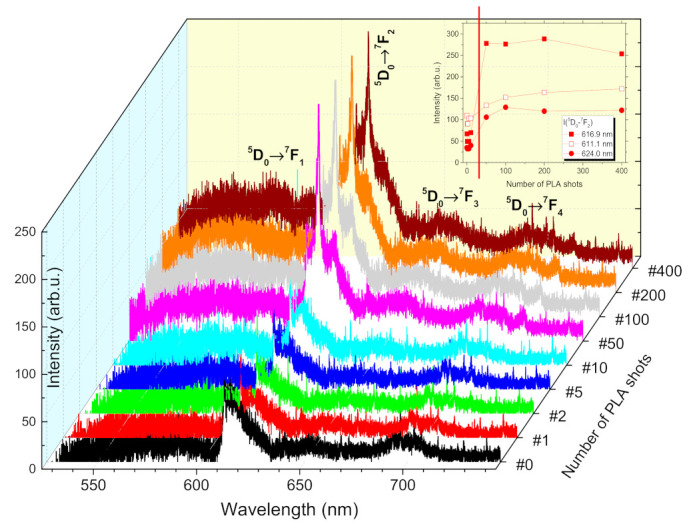
PL emission spectra of TiO_2_:Eu thin film, dependence on the number of PLA shots.

**Figure 7:**
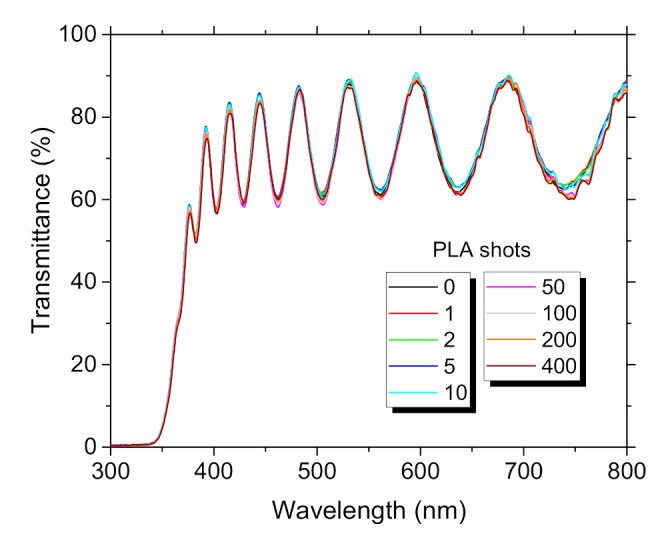
Transmittance spectra TiO_2_:Eu thin film, dependence on the number of PLA shots.

The dependence of optical properties of TiO_2_:Eu films, i.e., PL emission and transmittance spectra, on the number of PLA shots is presented in Figure 6 and Figure 7, respectively. The transmittance spectra underline the high quality of the film because any evident absorption in VIS that can be related to defects such as oxygen vacancies and a presence of Ti suboxides did not appear [28]. We could observe a weak broadened emission line of Eu^3+^ for the as-grown, which is characteristic for the amorphous phase [49]. The increase of Eu^3+^ PL intensity accompanied by sharpening lines of split ^5^D_0_→^7^F_2_ transition, as depicted in Figure 6, as well as a slight increase of transmittance (1.3%) are corelated to surface modification and gradual crystallization of TiO_2_ matrix. The significant increase of Eu^3+^ PL intensity (~5×) was detected at N_#_ = 50, where transmittance decreased and further remained constant. TiO_2_ nanocrystallites formed by PLA may act as sensitizers absorbing the excitation energy more efficiently [31,49]. We can recognize splitting ^5^D_0_→^7^F_2_ transition to peaks located at 611.1 nm, 614 nm, 616.9 nm, 624 nm, and 626.1 nm. The splitting may indicate Eu^3+^ located in D_2_ and S_4_ symmetry sites of TiO_2_ [49]. The slight decrease of Eu^3+^ PL intensity of ^5^D_0_→^7^F_2_ transitions at 616.9 nm and 624 nm after N_#_ = 200 can be attributed to the creation of well-crystalized anatase TiO_2_ matrix [28,50]. Because the intensity of ^5^D_0_→^7^F_1_ transition was weak, it made it impossible to conclude on its splitting. The ^5^D_0_→^7^F_0_ transition could not be followed at all, which may exclude Eu^3+^ occupying C_2v_ symmetry sites. Concerning transmittance, we must notice that its variation within PLA processing was generally rather weak. Its relative variation was less than 2%, which is slightly above the measurement error.

We did not obtain any shift in E_g_ value after PLA, as listed in Table 2. Similar values of E_g_~3.22 eV for TiO_2_ material [51] and anatase-like thin TiO_2_ films [52] were reported. There are reports of higher Eg~3.3 eV for TiO_2_:Eu films deposited by the spray pyrolysis technique. Our E_g_ values suggested we did not introduce defects in the films since lowering E_g_ from 3.03 eV to 2.52 eV was observed after aerodynamic levitated laser annealing of defect-rich (oxygen vacancies) TiO_2_ nanoparticles [53]. Lower E_g_~2.7 eV was also reported for TiO_2_:Eu films prepared by MAPLE, where E_g_ value shift was related to interstitial Eu doping [28].

**Figure 8:**
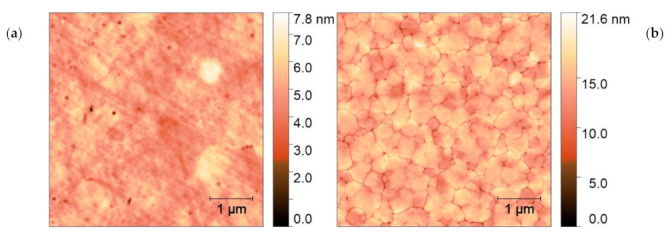
AFM surface images of TiO:Eu thin film (**a**) as deposited and (**b**) after PLA processing, 400 shots.

An AFM image of the film surface, shown in Figure 8, revealed clear evidence of crystallization of the film after PLA, as observed by XRD. The growth of grains is reflected by the increasing roughness, as depicted in Table 2.

**Figure 9:**
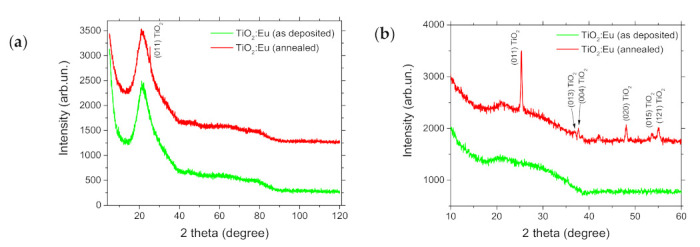
XRD patterns of TiO_2_:Eu thin film (as deposited and after PLA processing, 400 shots), (**a**) 2theta-omega and (**b**) grazing incidence scans.

XRD analyses, shown in Figure 9, revealed the amorphous character of as-grown film, while the PLA-treated film exhibited polycrystalline tetragonal anatase phase TiO_2_, as referred in the JCPDS Card NO: 96-500-0224 file. Peak positions and reflections’ planes derived from XRD patterns were 25.38° (011), 36.78° (013), 37.68° (004), 48.03° (020), 53.68° (015), and 55.08° (121). Crystallites’ size was derived using Sherrer’s formula and was ≈ 46.1 nm. Anatase TiO_2_ phase was also obtained by KrF PLA of nanoparticles at F_L_ = 34 mJ/cm^2,^ while at a higher F_L_ a rutile phase appeared [54]. The crystallization of divalent- or trivalent europium-containing oxide compounds or europium titanate [55] was not detected by XRD. The large mismatch in the ion radii of Eu^3+^ and Ti^4+^, 0.95 A and 0.68 A, respectively, may cause a crystal structural distortion and also make difficult the substitution of Ti^4+^ by Eu^3+^ within the anatase lattice [31].

### 3.3. Lu_2_O_3_:Eu

**Figure 10:**
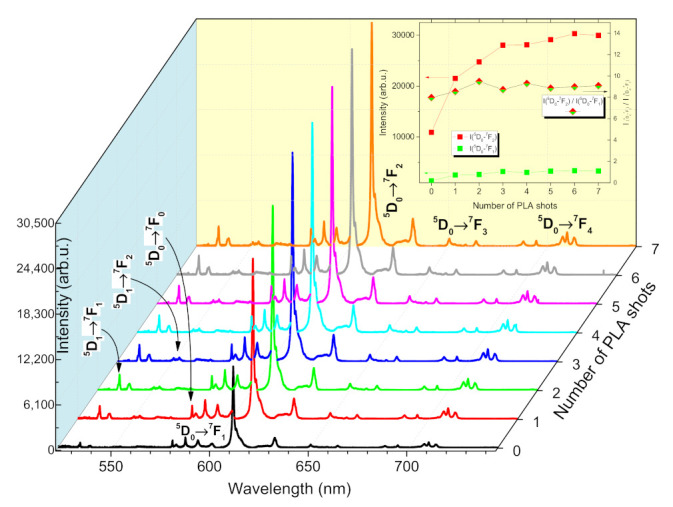
Emission spectra of Lu_2_O_3_:Eu thin film, dependence on the number of PLA shots.

The PL emission spectra of Lu_2_O_3_:Eu film, shown in Figure 10, exhibited a strong luminescence signal of Eu^3+^ attributed to transition ^5^D_0_→^7^F_j_ (j = 0–4) as well as a lower intensity signal from transition ^5^D_1_→^7^F_j_ (j = 1–2). The excitation efficiency by ArF laser at λ_exc_ = 193 nm profited from Lu_2_O_3_ host lattice broad absorption band centered around λ = 210 nm [40]. Let us notice that the appearance of ^5^D_1_→^7^F_j_ (j = 1–2) transitions is much less common in Eu^3+^-doped materials. These transitions could be observed in compounds with low-lattice phonon energy, e.g., Lu−O that leads to multiphonon relaxation [12,56]. They were also reported at films grown by chemical vapor deposition (CVD) [3]. Concerning ^5^D_0_→^7^F_j_ (j = 0–2) transitions, the PL spectrum revealed similar features as the spectrum (excited at λ_exc_ = 254 nm) of PLD-prepared Lu_2_O_3_:Eu film with 5% Eu content [27]. Transitions were composed of peaks at 580.2 nm for ^5^D_0_→^7^F_0_ (Eu^3+^ in the noncentrosymmetric C_2_ site [57]); 582.2 nm, 586.7 nm, 593.1 nm, and 600.3 nm for ^5^D_0_→^7^F_1_ (Eu^3+^ in the C_2_ and S_6_ (centrosymmetric) sites [40,58]); 611.0 nm, 612.6 nm, 624 nm, and 632 nm for ^5^D_0_→^7^F_2_ (Eu^3+^ exclusively in the C_2_ site [40,58]); 650.2 nm and 664 nm for ^5^D_0_→^7^F_3_; 708 nm, 710.1 nm, and 713.5 nm for ^5^D_0_→^7^F_4_; 533.4 nm for ^5^D_1_→^7^F_1_; and 553.6 nm for ^5^D_1_→^7^F_2_. The most intense peak observed at 611 nm is characteristic of the cubic phase of rare earth sesquioxides [25,32,40].

**Figure 11:**
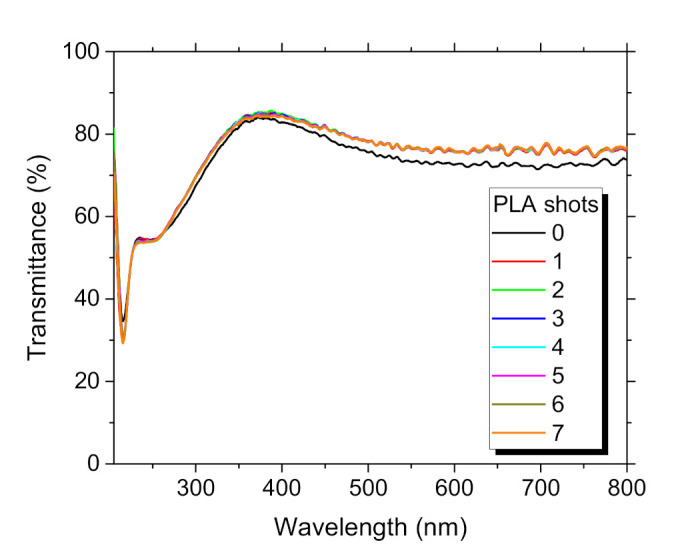
Transmittance spectra Lu_2_O_3_:Eu thin film, dependence on the number of PLA shots.

**Figure 12:**
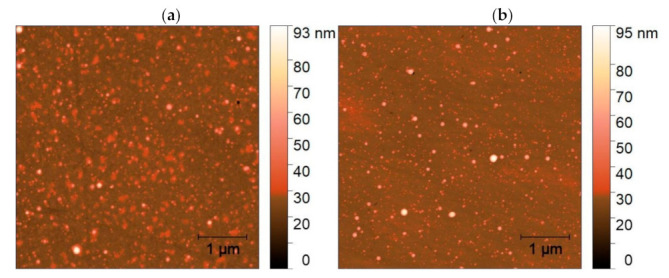
AFM surface images of Lu_2_O_3_:Eu thin film (**a**) as deposited and (**b**) after PLA processing, seven shots.

**Figure 13:**
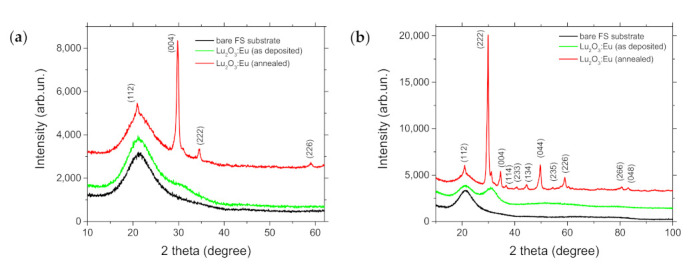
XRD patterns of Lu_2_O_3_:Eu thin film (as deposited and after PLA processing, seven shots), (**a**) 2theta-omega and (**b**) grazing incidence scans.

We noticed the most substantial change in the film optical properties, shown in Figure 10 and Figure 11, after the first PLA shot. The most substantial increase of Eu^3+^ intensity emission and optical transmittance was obtained. The E_g_ value shifted from 5.47 eV to 5.51 eV, where the latter values corresponded to those reported for evaporated Lu_2_O_3_ films [43,59]. This may be attributed to both surface and structure modifications (as observed by AFM and XRD in Figure 12 and Figure 13, respectively). The asymmetric ratio increased from 7.9 to 9.5 reached at N_#_ = 2, which is reflecting the site symmetry decreasing related with film structure modification. The asymmetric ratio value was then decreasing to 9 and not changing for N_#_ = 4–7. PLA led to the maximum enhancement of 2.8 times for Eu^3+^ intensity emission at 611.0 nm. The energy transfer from Eu^3+^ residing in S_6_ to Eu^3+^ in C_2_ may take place because the levels of Eu^3+^ in the site S_6_ lie above those for site C_2_. It was reported, based on following the ^5^D_0_→^7^F_1_/^5^D_0_→^7^F_0_ ratio, that the rate of the energy transfer may change with the crystallites’ sizes (it is lower for larger crystallites) or because the location of Eu^3+^ in C_2_ site is more common for small crystallites [57,60]. We did not observe any considerable variation of this ratio within the PLA process, although the crystallites’ size varied, as confirmed by XRD.

AFM analyses, shown in Figure 12, revealed similar morphology for as-deposited and PLA-treated film with typical S_q_ = 3.7 nm. Slight surface smoothing was observed after PLA and S_a_ decreased from 2.04 nm to 1.48 nm.

A substantial improvement of the crystalline structure after PLA treatment was evidenced by XRD diffraction patterns, depicted in Figure 13. While as-deposited film was rather amorphous, the film revealed a polycrystalline structure after seven PLA shots. The estimated crystallites’ size was 10.9 nm. The similar XRD pattern was observed for Lu_2_O_3_:Eu films deposited by PLD at a substrate temperature higher than 400 °C [25] and by magnetron sputtering with thermal postdeposition treatment at 900 °C for 2 h [20]. The reflections in the XRD pattern can be assigned to a body-centered cubic (I 21 3) structure of Lu_2_O_3_, according to JCPDS Card NO: 96-101-0596 file. The Eu_2_O_3_ phase was not detected in the patterns, confirming the Eu dopant was substituting Lu in Lu_2_O_3_ host, which was supported by the closed values of the ionic radii of 0.95 Å and 0.85 Å for Eu^3+^ and Lu^3+^, respectively [25]. The broad hump around 2θ = 21° is characteristic for amorphous fused silica substrate.

## 4. Conclusions

The in situ monitoring system of optical properties (photoluminescence and transmission) at pulsed laser annealing processing of Eu-doped oxide thin films was demonstrated. Eu-doped ZnO, TiO_2_, and Lu_2_O_3_ films were fabricated by PLD at room temperature. The system allowed us to find quickly the optimal PLA conditions (ArF laser at λ = 193 nm, fluence, and number of PLA shots) for maximizing Eu^3+^ photoluminescence. The Eu^3+^ emission enhancement of 4.1×, 5× and 2.8×, after 800, 400, and 7 PLA shots, for ZnO, TiO_2_, and Lu_2_O_3_ matrix was obtained, respectively. Transmission was markedly modified at Lu_2_O_3_:Eu film, where also *E*_g_ increased to 5.51 eV. On the other hand, ZnO:Eu and TiO_2_:Eu films exhibited lowering *E*_g_ to 3.25 eV and 3.23 eV, respectively. TiO_2_:Eu and Lu_2_O_3_:Eu as-deposited films exhibited an amorphous character that was modified by PLA to anatase and cubic nanocrystalline phase, respectively. In the case of ZnO:Eu, the application of PLA induced rather surface smoothening-related grains’ coalescence.

## Figures and Tables

**Table 1:** The deposition and laser annealing conditions.

Material	Fluence PLD (J⋅cm^−2^)	O_2_ Pressure (Pa)	Deposition Time (min)	Thickness (nm)	Fluence PLA (J⋅cm^−2^)
ZnO:Eu (1% at. Eu)	5	15	8.3	840–1000	175
TiO_2_:Eu (1% at. Eu)	9	1	25	800	175
Lu_2_O_3_:Eu (3% at. Eu)	5	2.5	20	110-150	150

**Table 2:** Properties, modifications after PLA.

Material	Eu^3+^ (^5^D_0_*→*^7^F_2_) PL Emission Enhancement	Band Gap *E*_g_ (eV)		Roughness *S* a *S* q (nm)	
	**PLA**	**As Deposited**	**PLA**	**As Deposited**	**PLA**
ZnO:Eu	4.1×	3.28	3.25	14.6 18.1	3.5 4.6
TiO_2_:Eu	5×	3.24	3.23	0.5 0.6	1.4 1.8
Lu_2_O_3_:Eu	2.8×	5.47	5.51	2.0 3.8	1.5 3.7

## Data Availability

The data that support the findings of this study are available from the corresponding author upon reasonable request.
